# Minimum number of clusters and comparison of analysis methods for cross sectional stepped wedge cluster randomised trials with binary outcomes: A simulation study

**DOI:** 10.1186/s13063-017-1862-2

**Published:** 2017-03-09

**Authors:** Daniel Barker, Catherine D’Este, Michael J. Campbell, Patrick McElduff

**Affiliations:** 1 School of Medicine and Public Health, Faculty of Health, University of Newcastle, Newcastle, NSW Australia; 2 National Centre for Epidemiology and Population Health, Research School of Population Health, Australian National University, Canberra, Australia; 3 Medical Statistics Group, ScHARR, University of Sheffield, Sheffield, UK; 4 Health Policy Analysis Pty Ltd, Sydney, NSW Australia; 5 CCEB, University of Newcastle, HMRI Building, Level 4 West, University Drive, Callaghan, NSW 2308 Australia

**Keywords:** Stepped wedge, Cluster randomised, Simulation study, Statistical analysis, Cross sectional

## Abstract

**Background:**

Stepped wedge cluster randomised trials frequently involve a relatively small number of clusters. The most common frameworks used to analyse data from these types of trials are generalised estimating equations and generalised linear mixed models. A topic of much research into these methods has been their application to cluster randomised trial data and, in particular, the number of clusters required to make reasonable inferences about the intervention effect. However, for stepped wedge trials, which have been claimed by many researchers to have a statistical power advantage over the parallel cluster randomised trial, the minimum number of clusters required has not been investigated.

**Methods:**

We conducted a simulation study where we considered the most commonly used methods suggested in the literature to analyse cross-sectional stepped wedge cluster randomised trial data. We compared the per cent bias, the type I error rate and power of these methods in a stepped wedge trial setting with a binary outcome, where there are few clusters available and when the appropriate adjustment for a time trend is made, which by design may be confounding the intervention effect.

**Results:**

We found that the generalised linear mixed modelling approach is the most consistent when few clusters are available. We also found that none of the common analysis methods for stepped wedge trials were both unbiased and maintained a 5% type I error rate when there were only three clusters.

**Conclusions:**

Of the commonly used analysis approaches, we recommend the generalised linear mixed model for small stepped wedge trials with binary outcomes. We also suggest that in a stepped wedge design with three steps, at least two clusters be randomised at each step, to ensure that the intervention effect estimator maintains the nominal 5% significance level and is also reasonably unbiased.

**Electronic supplementary material:**

The online version of this article (doi:10.1186/s13063-017-1862-2) contains supplementary material, which is available to authorized users.

## Background

Cluster randomised trials (CRTs) have become commonplace in health-related research and have been applied to a wide range of interventions [1]. The defining feature of the CRT is the randomisation of groups of individuals (termed clusters hereafter) rather than individual randomisation. As a result of this feature, the outcomes for individuals within clusters are likely to be correlated and the statistical analysis must take this into account.

Stepped wedge CRTs (SW-CRTs) are a variant of CRTs in which all clusters begin in the control phase and end in the intervention phase, and different clusters switch from control to intervention at different time points in random order. The stepped wedge design has been employed with increasing frequency in recent years and a recent systematic review reported that the number of SW-CRTs publications had increased substantially since 2010 [2].

In addition to adjusting for clustering, the analysis of a SW-CRT must consider the potential confounding effect of time, which is an unavoidable product of the study design if there is change in the outcome over time independent of the intervention effect [3–7]. For example, if the incidence of a disease decreases over time independently of the intervention, then failure to adjust for time would result in a biased estimate of the treatment effect. This is because randomisation into a SW-CRT causes an association between the intervention and time via an increase in the number of clusters allocated to the intervention as the study progresses. Despite the need to include time as a covariate defined *a priori* in the main analysis of a SW-CRT, there has been little investigation into the impact of adjusting for time on the power of the study, with the exception of the work by Baio et al. [8]. It has been suggested that a SW-CRT will require fewer clusters than a parallel CRT [7, 9–11] and recent literature has shown that this is indeed the case when the intra-cluster correlation coefficient (ICC) is high and clusters are large [12]. This is perhaps one of the reasons for the increased use of the SW-CRT in recent years [2, 13].

The problems with the different methods of analysis when there are few clusters in a CRT are well documented. For example, the robust variance estimator (RVE) used in the generalised estimating equation (GEE) framework underestimates the variance when there are fewer than 40 clusters [14–17] and it is recommended that generalised linear mixed models (GLMMs) have at least 10 clusters to properly estimate random effects [18]. In contrast, the minimum number of clusters required for reasonably unbiased estimation of the intervention effect in SW-CRTs is under-explored. This is especially pertinent because 45% of SW-CRTs in the review by the authors of this manuscript [13] had fewer than ten clusters. Furthermore, we noted in our review of this work that 62% of SW-CRTs used a binary measure as the primary outcome.

Arising from this are two logical questions. First, which of the currently used methods of analysis is best for an SW-CRT with a binary outcome when the number of clusters is small? Second, what is the minimum number of clusters required for the consistent and unbiased estimation of the treatment effect in a SW-CRT? To help answer these questions we present a simulation study for a SW-CRT with a binary outcome, with the simulation study designed according to the guidelines provided by Burton et al. [19]. The study is organised into three parts: first we describe in detail the simulation procedures and methods for generating the data based on a beta binomial model, second we describe the scenarios under investigation and third we briefly review the candidate methods that are most often employed to analyse the data from “standard” parallel CRTs or SW-CRTs. We then present the results of these simulations with emphasis on the bias, type I error rate and power for each method. Finally we discuss the implications of these results with special reference to smaller SW-CRTs.

## Methods

### Simulation aims

The goal of the simulation study was to examine the minimum number of clusters needed for a SW-CRT with a binary outcome by comparing the bias, type I error rate and power of commonly used analysis techniques under a range of plausible scenarios.

### Simulation procedures

Data sets were simulated based on a SW-CRT with three different intervention time points (steps) and four measurement periods. Prior to the first measurement period all the clusters are in the control condition and prior to each subsequent measurement period a third of the clusters are randomly selected to switch from the control to the intervention condition, until at the fourth measurement period all clusters are in the intervention condition. For each simulated data set the intervention effect was estimated by all the candidate analysis methods and their performance compared. The candidate methods and each of the scenarios conditions are described below.

SAS 9.3 software was used to generate and analyse the data. Where random number generation is required the ‘RAND’ series of functions was used. To create independent data sets for each replication, the starting seed was chosen such that no two replications contain repeats, which for the RAND functions occur after every 2^19937^–1 generations. Simulations that produced data sets in which there were no events of interest in any of the clusters when they were in the control condition were discarded and rerun with new starting seeds. Similarly simulations that produced data sets in which there were no events of interest in any of the clusters when they were in the intervention condition were also discarded and rerun. In practice, discarded data sets were a very rare occurrence.

### Methods for generating data

Consider a cross-sectional SW-CRT with four measurement periods and three steps for comparing a new intervention to a control condition. Let *Y*_*ijk*_ be a binary outcome with *Y*_*ijk*_ = 1 defining the event of interest and *Y*_*ijk*_ = 0 otherwise for the *i* ^th^ subject (*i* = 1,…,*N*) at the *j* ^th^ time (*j* = 0,1,2,3) in the *k* ^th^ cluster (*k* = 1,…,*M*). Let *X*_*jk*_ be the treatment indicator (1 = intervention; 0 = control) for the *k* ^th^ cluster at the *j* ^th^ time.

The first step in generating the data was to randomly sample the ‘true’ (i.e. population) cluster proportions from a beta distribution. Therefore baseline cluster proportions (*p*_0*k*_) were selected such that:

$$ {p}_{0 k}\sim Beta\left( a, b\right) $$

To ensure the data are correlated at the cluster level with a fixed ICC (*ρ*) and mean (*μ*), the parameters *a* and *b* in the beta distribution were obtained by solving the simultaneous equations [20]:

$$ E\left[{p}_{0 k}\right]=\mu =\frac{a}{a+ b} $$

$$ \rho =\frac{1}{a+ b+1} $$

The post-baseline ‘true’ cluster proportions (*p*_*jk*_) were then generated such that:

$$ {p}_{jk}=\frac{e^{\beta_{\mathrm{o} k}+{\beta}_1{X}_{jk}+{\beta}_2 j}}{1+{e}^{\beta_{\mathrm{o} k}+{\beta}_1{X}_{jk}+{\beta}_2 j}} $$

where *β*_1_ is the log odds ratio of the intervention effect and *β*_2_ is the log odds ratio of the effect at time *j* + 1 compared to time *j*. The parameter *β*_0*k*_ is equal to the logit of the baseline cluster proportions (*p*_0*k*_):

$$ {\beta}_{0 k}= log\left(\frac{p_{0 k}}{1-{p}_{0 k}}\right) $$

The final step was to generate *n*_*jk*_ independent subjects in each cluster *k* at each time *j*, which we refer to as the cell size from here on. These subjects have outcomes *Y*_*ijk*_ generated according to a Bernoulli distribution with probability *p*_*jk*_:

$$ {Y}_{ijk}\sim Bernoulli\left({p}_{jk}\right) $$

Since this was a cross-sectional SW-CRT, repeated measurements were not made on the same subjects within a cluster and there was therefore no serial correlation at the level of the individual, as would be expected in a cohort SW-CRT. For the purpose of generating our SW-CRT data, we have assumed that different measurement times from the same cluster are exchangeable.

### Scenarios under investigation

We simulated the data by expanding upon the procedures used by Ukoumunne et al. [21] to a SW-CRT scenario. We used a mean baseline control proportion *E*[*p*_0*k*_] of 0.1 and an intervention effect odds ratio of 2.25, which corresponds to a doubling of the proportion to 0.2. When a time trend was added to the data the value of the odds ratio for time *j* + 1 relative to time *j* was 1.227. We chose these values to represent a trial with a moderately large intervention effect, similarly to trials we reviewed previously [22–24], with the addition of a relatively smaller time trend. When a time trend was not required the odds ratio for time was set to one. We examined the scenarios in which the number of clusters randomised was 3, 6, 9, 18 and 36. These numbers were chosen such that situations with very few clusters were represented and so that each “step” had the same number of clusters switching from control to intervention condition. For each of these scenarios we generated data sets using cell sizes of 5, 10, 50 or 100 subjects and a baseline ICC of 0.01, 0.05 and 0.1 because most CRTs have an ICC within this range [25–27]. To estimate the type I error rate for each method, all the above simulations were repeated using an intervention effect odds ratio of one. In total, 240 scenarios using the three-step SW-CRT (referred to as scenario A hereafter) were investigated (5 number of clusters * 4 cluster size * 2 time effects * 3 ICCs * 2 intervention effects) with 2000 data sets being generated for each scenario so that estimated power and type I error rates have standard errors of approximately 0.009 and 0.005 respectively.

To expand on the range of scenarios we performed additional simulations on a SW-CRT with six steps, which we shall refer to as scenario B below. To keep the number of clusters divisible by the number of steps we chose 6, 12, 18 and 36 clusters. For each of these we used cell sizes of 5, 10, 25 or 50 and a baseline proportion of 0.2. To simulate a trial with weaker intervention and time effects, we used an intervention effect odds ratio of 1.33 and a time effect odds ratio of 1.03.

### Review of candidate methods

The candidate methods were chosen because of their widespread application to the analysis of SW-CRTs [28]. Methods included logistic regression within a GEE framework, logistic regression within a GLMM framework and logistic regression with cluster (*k*) included as a fixed effect, which from this point onwards shall be referred to as the fixed effects method. As suggested by Hussey and Hughes we also used a linear mixed model (LMM) based on summary data (i.e. mean probability) from each cluster at each time point [29].

### Generalised Linear Mixed Model (GLMM) approach

GLMMs are an extension to generalised linear models (GLMs) for analysing correlated data [30]. The term mixed arises because these models estimate both fixed effects, which are the deterministic part of the model forming the regression line and random effects, which in the context of CRTs estimate the stochastic variation of individual clusters around the conditional mean of the clusters.

The GLMM for the binary responses in the simulated data is:

$$ logit\left( E\left[{Y}_{jk}\right]\right)= log\left(\frac{p_{jk}}{1-{p}_{jk}}\right)={\beta}_0+{a}_{0 k}+{\beta}_1{X}_{jk}+{\beta}_2 j $$

where *a*_0*k*_ is a normally distributed random intercept at the level of the cluster. Gauss-Hermite quadrature with four quadrate points was used to approximate the model likelihood function. The null hypothesis for fixed effects parameters from these models was tested using a Wald test compared to a *t* distribution where the degrees of freedom were calculated using the containment approximation [31], which is the default method in SAS PROC GLIMMIX. We note here that compared to the data generation method, which simulated baseline cluster probabilities from a beta distribution, this model is miss-specified since it assumes the random intercept is normally distributed. We did this because in practice the true baseline distribution is likely to be unknown and most researchers will fit a model that assumes the random intercept will have a normal distribution. We would argue that in many situations when the outcome is binary and there is a real difference between clusters at baseline, the distribution of the true cluster proportions is just as likely to be from a beta distribution as it is to be from a normal distribution and therefore we were interested in how the model performed despite this limitation [13].

### Generalised Estimating Equation (GEE) approach

The GEE framework to GLM was first introduced by Liang and Zeger in 1986 [32]. Since then it has become a popular choice for the analysis of data from CRTs and longitudinal studies [33]. Unlike GLMMs, which model the variance and covariance arising from correlated data directly, the GEE method primarily aims to model the population average while accounting for the correlation indirectly. Variance estimates can either be model based, where the covariance structure is specified by the user, or utilise the RVE in addition to this. One advantage of the RVE is that it converges to the correct value when there are a sufficient number of clusters even when the correlation structure is miss-specified [34]. However, it is possible to improve the model efficiency (and hence require fewer clusters) by correctly specifying the underlying correlation structure [35].

For every simulated data set we applied the following GEE model:

$$ logit\left( E\left[{Y}_{jk}\right]\right)= log\left(\frac{p_{jk}}{1-{p}_{jk}}\right)={\beta}_0+{\beta}_1{X}_{jk}+{\beta}_2 j $$

For this mean model, estimation of the parameters and their variances utilised an exchangeable working correlation structure. P-values for individual parameters are based on the Wald test and were calculated using the standard normal distribution, which is the default in SAS PROC GENMOD when a repeated statement is used.

Diggle et al. [35] showed that the population-level effect that is estimated by a marginal model, such as the GEE above, will be closer to the null than the cluster specific effect estimated by a GLMM, such as model (1). This makes it difficult to compare GEE estimates with the other methods because they are fundamentally estimating different parameters. Neuhaus et al. [36] showed that for a binary outcome, the estimate from a conditional model is different from the estimate from a marginal model by a factor of 1–ICC. For the purposes of comparison between methods, we therefore estimate the cluster-specific estimate from model (2) as:

$$ {\widehat{\beta}}_1^{*}=\frac{{\widehat{\beta}}_1}{1-\rho} $$

### Fixed effects model specification

This method involves fitting a GLM with a fixed effect for cluster. This fixed effect method is not generally considered a good model for CRT data because the variance is underestimated unless the clusters sampled are the only clusters that exist [37]. However, we included this as a candidate method because it has been used to analyse recent SW-CRTs [38–42].

The general model for the individual binary responses in the simulated data is written as:

$$ logit\left( E\left[{Y}_{jk}\right]\right)= log\left(\frac{p_{jk}}{1-{p}_{jk}}\right)={\beta}_0+{\beta}_1{X}_{jk}+{\beta}_2 j+{\beta}_3 I\left( k=2\right)+\cdots +{\beta}_{M+1} I\left( k= M\right) $$

Null hypotheses of parameters in these models were assessed using the default method is SAS PROC GENMOD, which is a Wald test compared to a chi-squared distribution. *I*(*k* = *M*) is an indicator variable for cluster *M,* taking the value 1 if *k* = *M* and 0 otherwise*.*

### Cluster summaries model specification

The cluster summaries approach usually involves first calculating the cluster mean and then performing a t-test of those means to compare trial arms. In the context of SW-CRTs Hussey and Hughes [29] proposed that the proportion of “successes” ${\pi}_{jk}=\frac{{\displaystyle \sum {Y}_{ijk}}}{n_{jk}}$ for each cluster at every time be calculated and then modelled using a linear mixed model (LMM). For the simulated data we fit the following model:

$$ {\pi}_{jk}={\beta}_0+{a}_{0 k}+{\beta}_1{X}_{jk}+{\beta}_2 j $$

The null hypothesis for the fixed effects parameters in these models was also tested using a Wald test compared to the *t* distribution with the default containment degrees of freedom approximation [31]. An important difference between this model and the models from the other candidate methods is that parameter estimates from (*4*) are interpreted as risk differences whereas the other three candidate methods all estimate log odds ratios.

### Method of time adjustment

In general the approach to adjusting for time trends in a SW-CRT is to treat time as a categorical variable. In models (1) to (4) above this amounts to replacing *β*_2_ *j* with a series of indicator variables for each time *j* > 0. For example, model (1) fitted to scenario A would become:

$$ logit\left( E\left[{Y}_{jk}\right]\right)= log\left(\frac{p_{jk}}{1-{p}_{jk}}\right)={\beta}_0+{a}_{0 k}+{\beta}_1{X}_{jk}+{\beta}_2 I\left( j=1\right)+{\beta}_3 I\left( j=2\right)+{\beta}_4 I\left( j=3\right) $$

For the sake of simplicity we have assumed in both the generation and analysis of the data that the time trend is linear. In addition to examining models (1) to (4), we also examine the bias of these approaches when no attempt is made to adjust for time in the modelling approach, i.e. models (1) to (4) excluding the *β*_2_ *j* term.

### Estimates of interest and evaluation criteria

Since the primary goal of the cluster RCT is to estimate the intervention effect, the estimate of interest will be the intervention effect parameter and its associated p-value. For models (1) to (3), we calculated the bias as the estimated log odds ratio minus the true log odds ratio ${\widehat{\beta}}_1-{\beta}_1$ and the per cent bias as $\frac{{\widehat{\beta}}_1-{\beta}_1}{\beta_1}\times 100$ to assess how accurately the models estimate the intervention effect. For the cluster summary method the bias was calculated as the estimated risk difference minus the true risk difference (0.1 for scenario A and 0.05 for scenario B). In all comparisons we used a significance level corresponding to 5%; therefore the type I error rate was calculated as the proportion of *p*-values that were less than 0.05 in the scenarios where the intervention effect was set to null. The power was calculated as the proportion of *p*-values that are less than 0.05 for the scenarios where an intervention effect was present.

## Results

### Adjusting for time

**Fig. 1:**
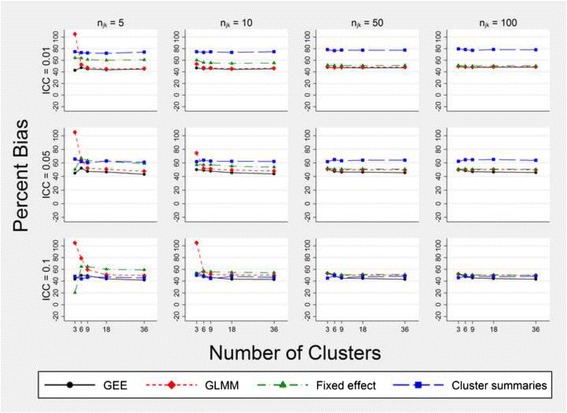
Per cent bias in the intervention effect estimate $\left({\widehat{\beta}}_1\right)$ for models that fail to adjust for time. Estimates are obtained from fitting models (1) to (4) without the time effect. Simulated data have three steps: a cell size equal to *n*_*jk*_, a true intervention effect odds ratio of 2.25 and a time effect odds ratio of 1.227

To examine the consequence of failing to adjust for time when a true time effect is present, Fig. 1 (and Additional file 1) present the results of fitting models (1) to (4) without the time covariate [i.e. incorrectly assuming (*β*_2_ = 0)]. These figures show that all methods of analysis are biased in every scenario when the time effect is ignored.

### Bias

**Fig. 2:**
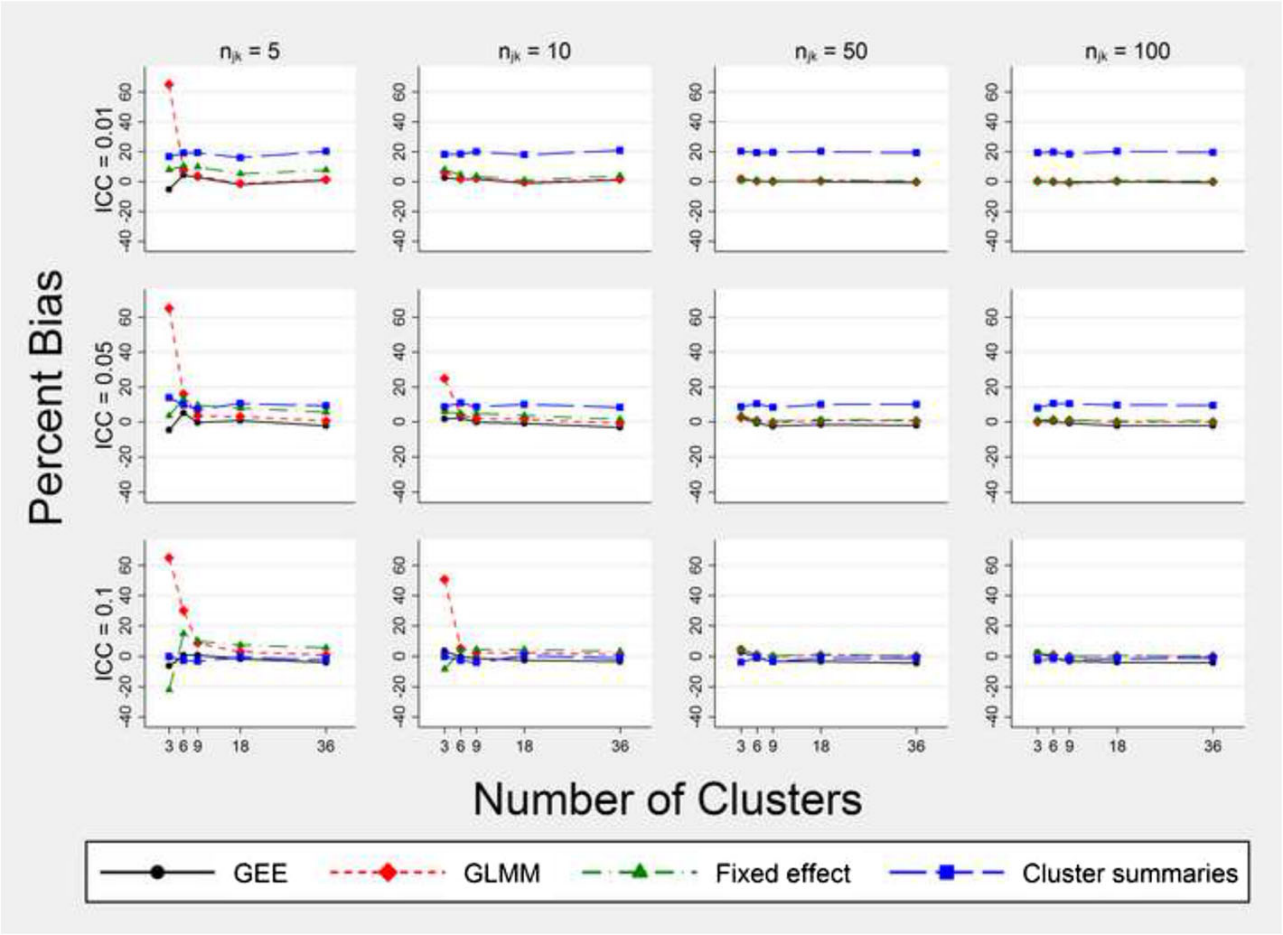
Per cent bias in the intervention effect estimate $\left({\widehat{\beta}}_1\right)$ for models that correctly adjust for time. Estimates are obtained from fitting models (1) to (4). Simulated data have three steps, a cell size equal to *n*_*jk*_, a true intervention effect odds ratio of 2.25 and a time effect odds ratio of 1.227

All intervention effect estimates were normally distributed; for the GEE, the GLMM and the fixed effects model this was on the logit scale and for the cluster summaries method this was on the proportion scale. When the models did correctly adjust for time, the bias associated with a particular method varied depending on the number of individuals within a cluster, the number of clusters and the magnitude of the ICC (Fig. 2, Additional file 2). For scenario A the approach using cluster summary statistics had approximately 20% bias when the ICC was 0.01 irrespective of the number of clusters but improved with an increasing level of ICC. In scenario B this method had a slight positive bias when the ICC was 0.01, which again reduced with increasing ICC, until it was consistently underestimating the intervention effect when the ICC was 0.1 (Additional file 2). The GLMM approach had a large positive bias when there were only three clusters and a cell size of 10 or less but became the most consistent method for unbiased estimation in both scenarios A and B when there were more than six clusters (Fig. 2, Additional file 2). The fixed effects model exhibited similar bias to the GLMM except that the former was more biased when the cell size was small. The GEE approach was the least biased of all methods when there were only three clusters, but when the ICC was 0.05 or greater, the GEE slightly underestimated the true intervention effect for larger cell sizes and greater numbers of clusters (Fig. 2). For scenario B, GEE bias was almost identical to GLMM bias (Additional file 2).

### Type I error rate

**Fig. 3:**
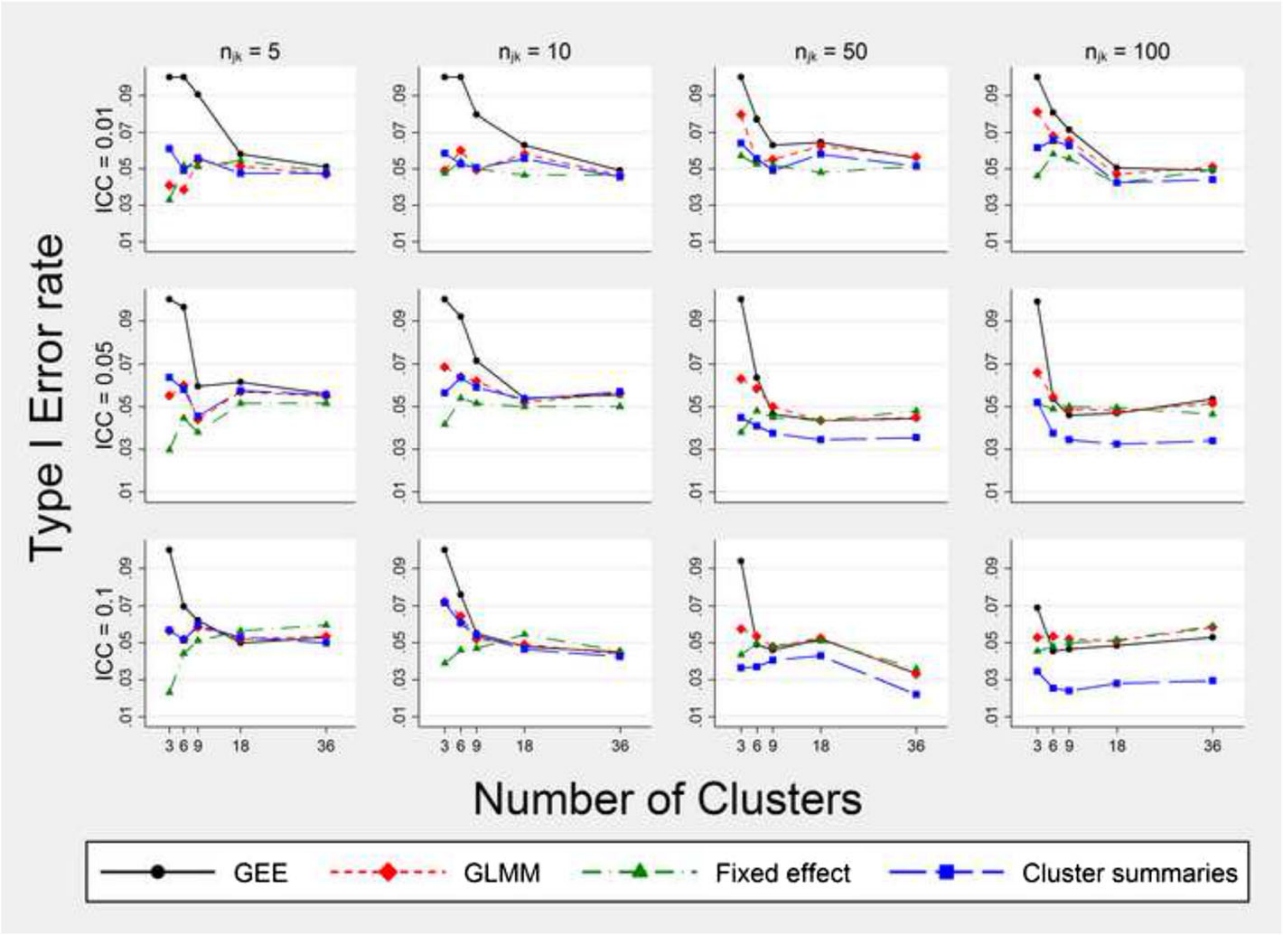
Type I error rate in the intervention effect estimate $\left({\widehat{\beta}}_1\right)$ for models that correctly adjust for time. Estimates are obtained from fitting models (1) to (4). Simulated data have three steps, a cell size equal to *n*_*jk*_, a true intervention effect odds ratio of 1 and a time effect odds ratio of 1.227

In general the type I error rate for all methods improved toward the nominal 5% level as the number of clusters and the cell size increased (Fig. 3, Additional file 3). However, for scenario A, the cluster summaries method was overly conservative regardless of the number of clusters or the cell size when the ICC was 0.05 or more. For scenario B this over-conservative tendency disappeared (Additional file 3). In both simulated SW-CRTs the GEE suffered from an inflated type I error rate when the number of clusters was nine or fewer, particularly when the cell size was small. Compared to the GEE, the GLMM had a comparatively less inflated type I error rate (at worst 8% when there only 3 clusters) but for both scenarios A and B the GLMM was most anticonservative when the ICC was 0.01, there were six clusters or fewer and the cell sizes were 25 or more. For most scenarios the fixed effects model had a type I error rate of close to 5% but when there were only three clusters and the cell size was five it was conservative.

### Power

**Fig. 4:**
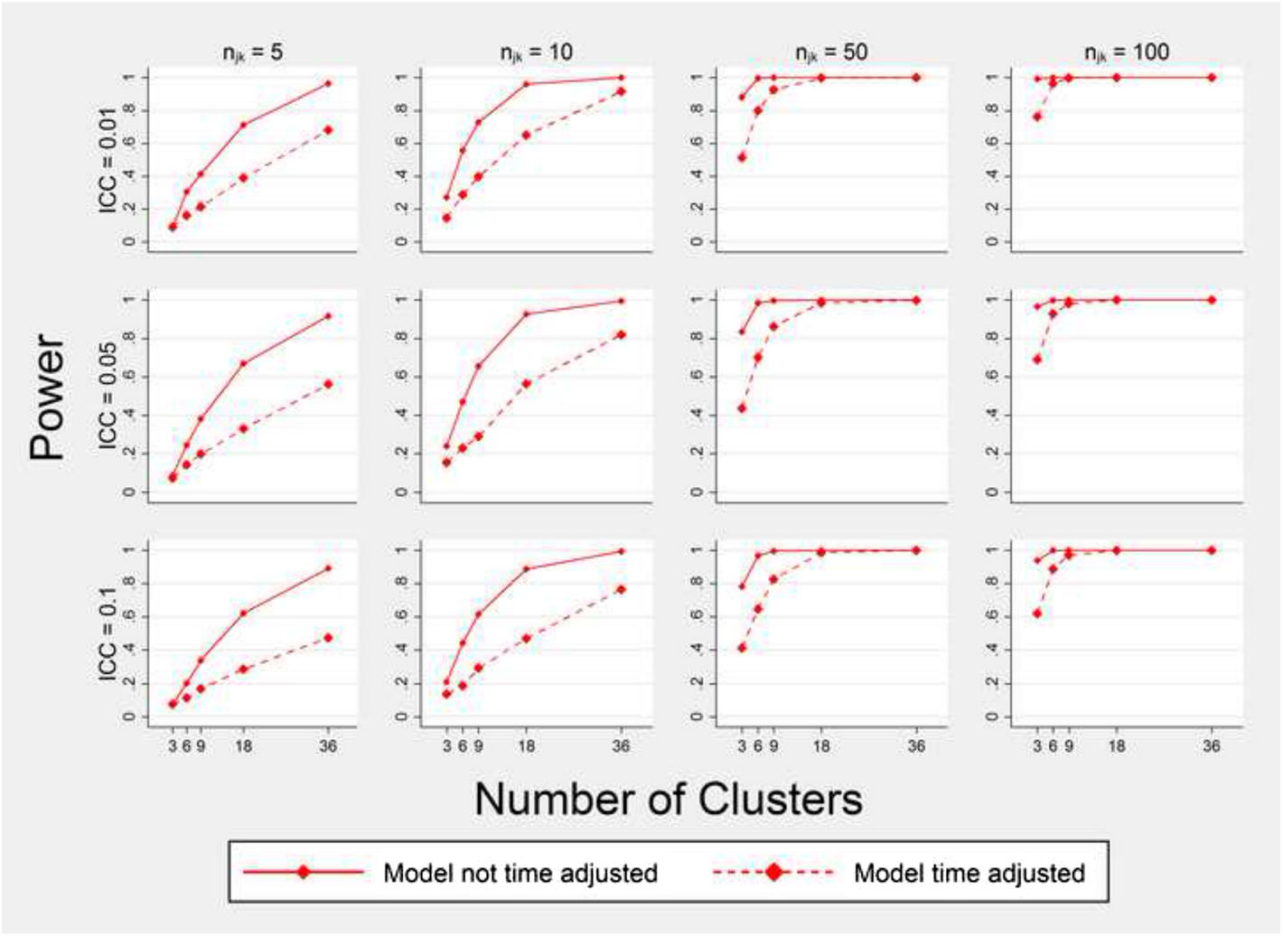
Power to detect the true intervention effect using a GLMM with and without adjustment for time. Estimates are obtained from fitting model (1) with and without time as a covariate. Simulated data have three steps, a cell size equal to *n*_*jk*_, a true intervention effect odds ratio of 2.25 and a time effect odds ratio of 1. Both models shown maintained a type I error rate of approximately 5%

Adjusting for time when no time effect is present leads to large losses in power for all of the analysis methods, irrespective of the ICC. Figure 4 demonstrates this for only the GLMM, but all methods showed a similar pattern.

The GEE and GLMM were the most powerful methods for most of the scenarios we simulated, reaching 80% and 90% power with fewer clusters and fewer subjects than the other methods (Table 1, Additional file 4). For scenario A, the fixed effects model compared favourably to the GEE and GLMM when the cell size and ICC were large. However, when the cell size was ten or fewer and the ICC was 0.05 or less, it was the least powerful analysis approach.

**Table 1:** Power to detect an intervention effect (OR = 2.25) in scenario A with different methods of analysis

*ICC*	*k*	*n*_*jk*_	True time effect OR = 1				True time effect OR = 1.227			
			GEE	GLMM	Cluster summaries method	Fixed effects model	GEE	GLMM	Cluster summaries method	Fixed effects model
0.01	3	100	0.806	0.765	0.657	0.735	0.858	0.828	0.670	0.801
	6	50	0.813	0.802	0.740	0.737	0.866	0.852	0.785	0.812
		100	0.971	0.965	0.929	0.955	0.979	0.980	0.945	0.969
	9	50	0.929	0.926	0.907	0.893	0.948	0.947	0.920	0.930
		100	0.998	0.998	0.993	0.996	0.999	0.999	0.995	0.998
	18	10	0.665	0.653	0.632	0.535	0.736	0.724	0.705	0.583
		50	0.997	0.998	0.995	0.989	0.999	0.999	0.999	0.999
	36	5	0.690	0.683	0.675	0.549	0.773	0.762	0.747	0.625
		10	0.918	0.916	0.908	0.806	0.953	0.953	0.947	0.874
0.05	3	100	0.698	0.690	0.464	0.684	0.734	0.733	0.401	0.750
	6	50	0.697	0.702	0.590	0.695	0.764	0.766	0.563	0.749
		100	0.925	0.929	0.810	0.923	0.962	0.966	0.758	0.961
	9	50	0.859	0.863	0.782	0.860	0.900	0.904	0.755	0.899
		100	0.984	0.983	0.951	0.982	0.996	0.995	0.934	0.995
	18	50	0.987	0.986	0.978	0.986	0.998	0.999	0.985	0.998
	36	10	0.826	0.820	0.807	0.778	0.874	0.871	0.848	0.832
0.1	3	100	0.597	0.619	0.320	0.621	0.659	0.685	0.229	0.692
	6	50	0.623	0.647	0.475	0.645	0.699	0.719	0.399	0.715
		100	0.871	0.888	0.673	0.892	0.919	0.936	0.534	0.932
	9	50	0.811	0.827	0.693	0.820	0.847	0.860	0.599	0.850
		100	0.968	0.971	0.865	0.970	0.979	0.984	0.765	0.984
	18	50	0.985	0.987	0.964	0.986	0.992	0.992	0.941	0.992
		100	1.000	1.000	0.999	1.000	1.000	1.000	0.986	1.000
	36	10	0.763	0.766	0.740	0.752	0.838	0.832	0.793	0.808

Only scenarios where at least one method had a power of between 0.7 and 1 are shown. Each estimate is based on 2000 simulations. All methods adjust for time in the model

### Convergence failures

For scenario B none of the models failed to converge and for scenario A there were no convergence failures when there were more than nine clusters. The GLMM and cluster summaries approach were the most reliable models with the most convergence failures occurring when there were only three clusters and a cell size of five (8 and 6 failed respectively). Table 2 shows that the GEE and fixed effects models failed to converge much more often in this same scenario (13% and 12.6% respectively) and in general.

**Table 2:** Number of convergence failures per 2000 simulations of scenario A

*ICC*	*k*	*n*_*jk*_	True time effect OR = 1				True time effect OR = 1.227			
			GEE	GLMM	Cluster summaries method	Fixed effects model	GEE	GLMM	Cluster summaries method	Fixed effects model
0.01	3	5	102	0	0	105	61	0	1	65
		10	7	0	0	7	2	0	1	2
	6	5	7	0	0	7	2	0	0	2
	9	5	1	0	0	1	0	0	0	0
0.05	3	5	193	5	1	182	133	2	0	141
		10	35	0	0	36	21	0	0	21
	6	5	10	0	0	11	8	0	0	8
	9	5	0	0	0	0	1	0	0	1
0.1	3	5	261	8	6	251	209	5	1	221
		10	85	3	0	85	63	0	0	65
		50	2	0	0	2	2	0	0	2
		100	1	0	0	0	2	0	0	2
	6	5	32	0	0	30	23	0	0	29
		10	1	0	0	1	3	0	0	3
	9	5	6	0	0	6	4	0	0	4

Only scenarios where at least one method had a convergence failure are shown. All methods adjust for time in the model

## Discussion

Out of all the analysis methods tested on our simulated data, we found that the GLMM approach with a random intercept was often the best analysis approach. For all values of the ICC it had a good type I error rate and bias characteristics when compared to the other methods while maintaining similar if not superior power despite the distribution of the random intercept being miss-specified. The GLMM is not without its problems though; in the scenarios we investigated, the bias was substantial when only three clusters with cell sizes of ten or fewer, especially when the ICC was higher and there were few subjects. When there were only 3 clusters and the cell sizes were 50 or more, the bias of the GLMM was much less, but the type I error rate was inflated. The major problems with the GEE are the inflated type I error rate and convergence failures when there are few clusters. If researchers wish to use a GEE when there are few clusters then we suggest that one of the corrections evaluated by Scott et al. [43] be considered. When there are six clusters there is some merit to the fixed effects modelling approach, which was less biased and more conservative than corresponding GLMMs when it converged.

In accordance with the literature on SW-CRT analysis [4–7] these results demonstrate that if there is no attempt to adjust for a time trend when one exists, the estimation of the intervention effect will be biased. While this bias will depend on the magnitude of the time trend, most often the presence and magnitude of any time trend will be unknown. When a time trend is adjusted for, it is more robust in general for it to be fitted as a categorical variable rather than as a continuous variable, which we have assumed for the sake of simplicity. The decision of how to adjust for time in the analysis can be informed by knowledge of the trial subject matter at hand; however, we note that current methods for calculating the power/sample size of an SW-CRT do so based on a model that adjusts for time as a categorical variable rather than a continuous one so that the type I error rate is correct [7, 29].

It is widely regarded that the SW-CRT is more powerful than a traditional cluster RCT [3, 7, 10, 11]. Although this has now been proven to be not universally the case [12], we suspect that this belief has contributed to the large number of stepped wedge studies with very few clusters. However, these same studies regularly use either a GEE or GLMM modelling approach for binary outcomes, which we have shown have at least one undesirable statistical property when there are few clusters. We also point out that these simulations reflect an ideal scenario where there are no missing data and the cluster sizes are equal. There is the distinct possibility that the number of clusters required will increase when the situation departs from these ideals or when the analysis increases in complexity, such as when additional random effects terms or interactions are added to the model.

There are also other problems with randomising very few clusters, which apply to SW-CRTs and CRTs alike. As Taljaard et al. point out, results from trials with few clusters may not be generalisable to wider populations [44]. A related concern is that the benefit of randomisation is potentially lost as the balance of known and unknown confounders depends on sufficient numbers of clusters being randomised [44, 45].

Our study was limited by several factors. Due to computing restraints, only 2000 data sets were simulated for each scenario and much more stable estimates could be obtained by using a larger number. The models we used were simple and their suitability for analysing small SW-CRTs varied. For example, the GEE we used was limited by the default settings in PROC GENMOD, which do not implement a degrees of freedom correction like the one the GLMM model benefited from. We also made the assumption that the correlation within a cluster is exchangeable. It is very possible that this correlation could in fact be autoregressive in some settings, in which case none of the analysis methods presented here would sufficiently control the type I error rate. Further research into this subject is warranted. Another distinct possibility is that the time trend is not linear, as was assumed above because the data were simulated as such. Fitting time as a categorical variable will be required in the event this assumption is not reasonable and in general modelling time this way will give an unbiased estimate of the intervention effect but may require more than six clusters. Further research is needed to determine whether the loss in power from such an approach is substantial.

## Conclusion

In summary we recommend that SW-CRTs with a limited number of clusters and binary outcomes should be analysed using a GLMM. Our strongest recommendation of all is that a cross-sectional SW-CRT with three steps should not randomise fewer than six clusters and that when few clusters are available there needs to be a large number of subjects per cluster per time.

## Additional files

Additional file 1: TIFF image, LZW compression. Per cent bias in the intervention effect estimate $\left({\widehat{\mathit{\ss}}}_1\right)$ for models that fail to adjust for time. Estimates are obtained from fitting models (1) to (4). Simulated data have six steps, a cell size equal to *n*_*jk*_, a true intervention effect odds ratio of 1.33 and a time effect odds ratio of 1.03. (TIF 269 kb)
Additional file 2: TIFF image, LZW compression. Per cent bias in the intervention effect estimate $\left({\widehat{\ss}}_1\right)$ for models that correctly adjust for time. Estimates are obtained from fitting models (1) to (4). Simulated data have six steps, a cell size equal to *n*_*jk*_, a true intervention effect odds ratio of 1.33 and a time effect odds ratio of 1.03. (TIF 267 kb)
Additional file 3: TIFF image, LZW compression. Type I error rate in the intervention effect estimate $\left({\widehat{\mathit{\ss}}}_1\right)$ for models that correctly adjust for time. Estimates are obtained from fitting models (1) to (4). Simulated data have six steps, a cell size equal to *n*_*jk*_, a true intervention effect odds ratio of 1 and a time effect odds ratio of 1.03. (TIF 279 kb)
Additional file 4: Power to detect intervention effect (OR = 1.33) in a six-step SW-CRT with different methods of analysis. Each estimate is based on 2000 simulations. All methods adjust for time in the model. (RTF 153 kb)
Additional file 5: Simulation code for scenario A. (DOCX 22 kb)
Additional file 6: Simulation code for scenario B. (DOCX 22 kb)

## Abbreviations

CRT: cluster randomised trial
GEE: generalised estimating equation
GLM: generalised linear model
GLMM: generalised linear mixed model
ICC: intra-cluster correlation coefficient
LMM: linear mixed model
RVE: robust variance estimator
SW-CRT: stepped wedge cluster randomised trial
