# Supplementary figures and images for: Minimum number of clusters and comparison of analysis methods for cross sectional stepped wedge cluster randomised trials with binary outcomes: A simulation study
Source: Trials. 2017 Mar 9;18:119. doi: 10.1186/s13063-017-1862-2 (PMC5345156; doi:10.1186/s13063-017-1862-2)

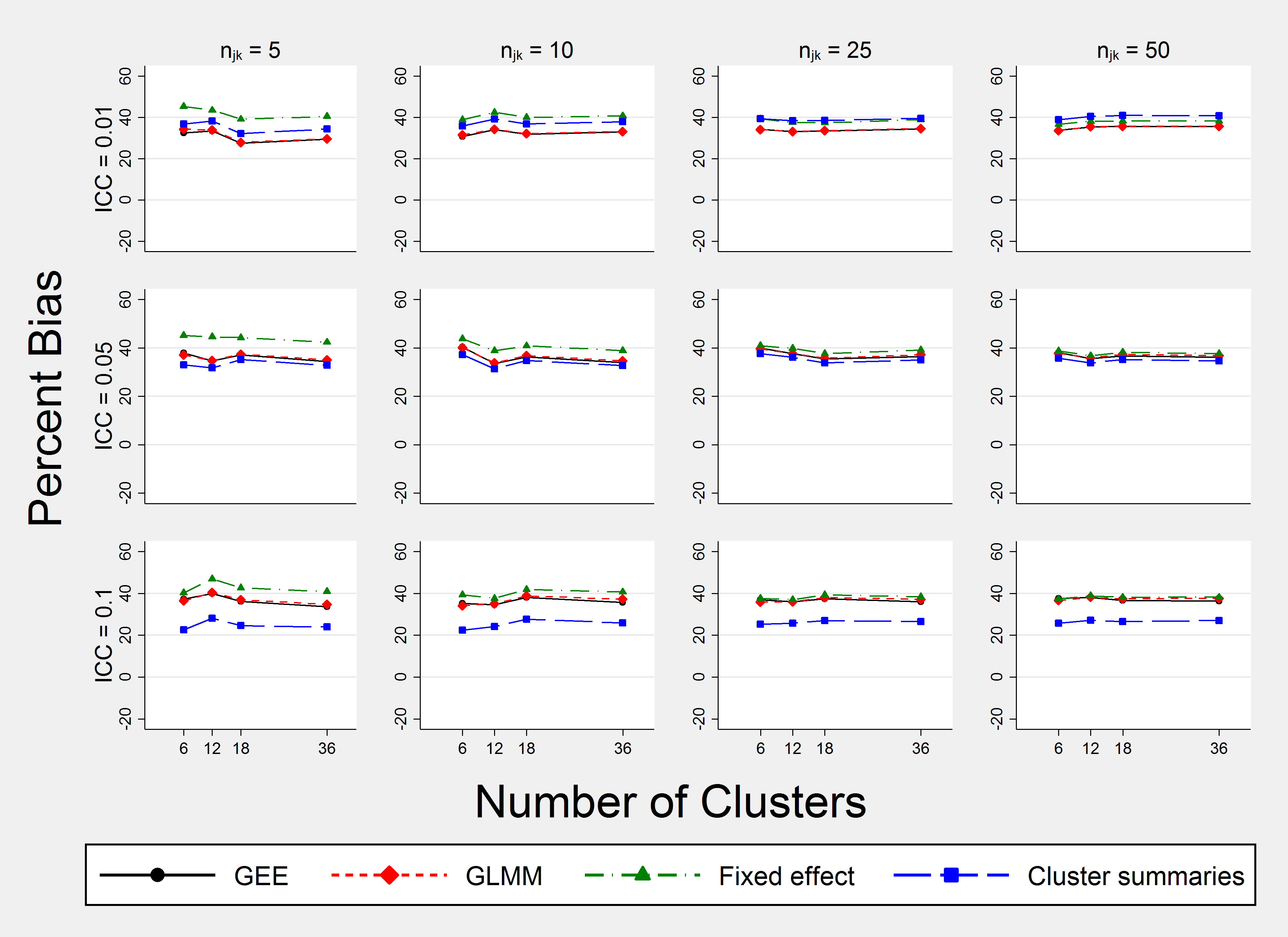

Supplement: Additional file 1: — TIFF image, LZW compression. Per cent bias in the intervention effect estimate $\left({\widehat{\mathit{\ss}}}_1\right)$ß^1 for models that fail to adjust for time. Estimates are obtained from fitting models (1) to (4). Simulated data have six steps, a cell size equal to n jk, a true intervention effect odds ratio of 1.33 and a time effect odds ratio of 1.03. (TIF 269 kb) [file 13063_2017_1862_MOESM1_ESM.tif]

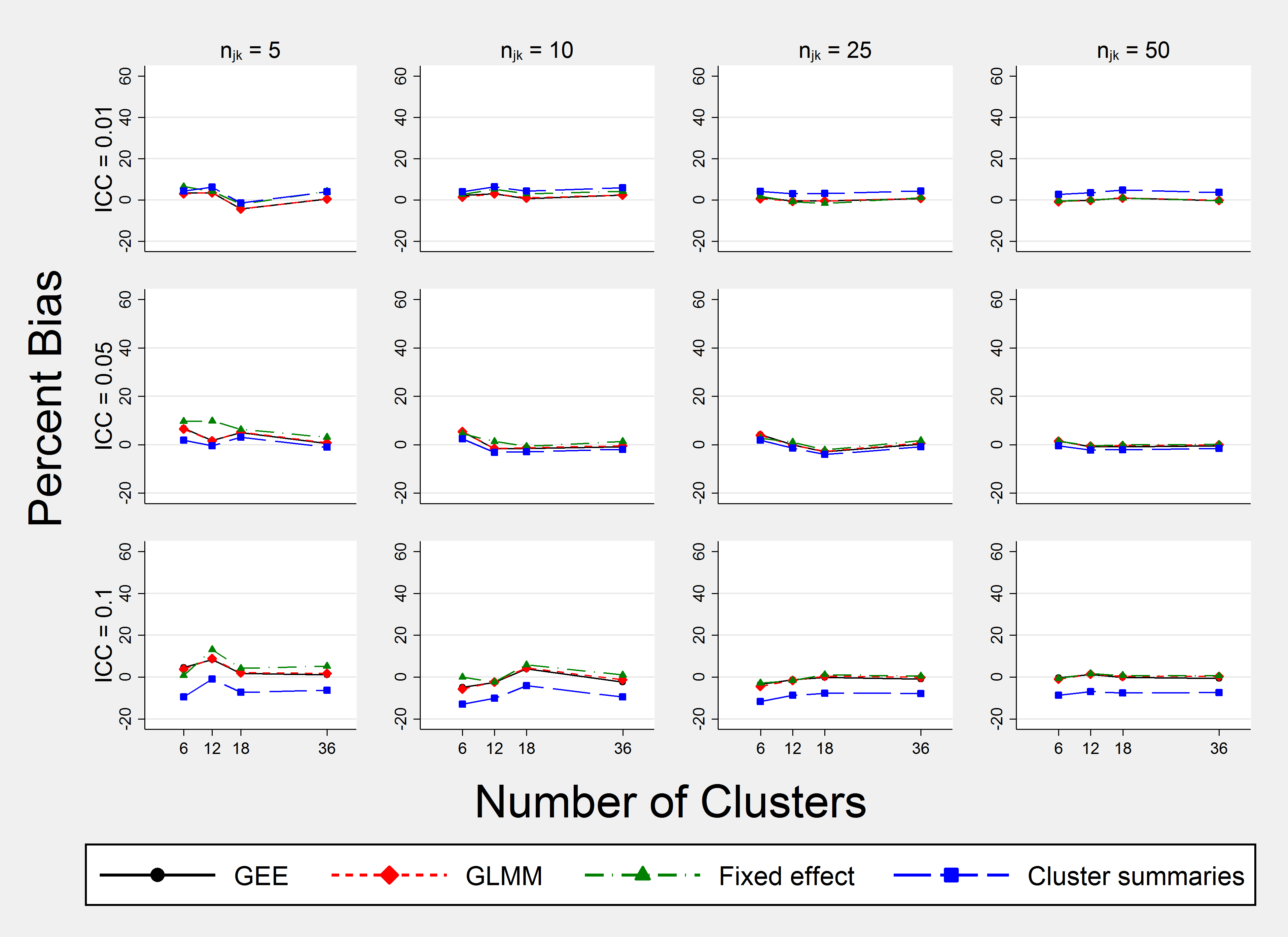

Supplement: Additional file 2: — TIFF image, LZW compression. Per cent bias in the intervention effect estimate $\left({\widehat{\ss}}_1\right)$ß^1 for models that correctly adjust for time. Estimates are obtained from fitting models (1) to (4). Simulated data have six steps, a cell size equal to n jk, a true intervention effect odds ratio of 1.33 and a time effect odds ratio of 1.03. (TIF 267 kb) [file 13063_2017_1862_MOESM2_ESM.tif]

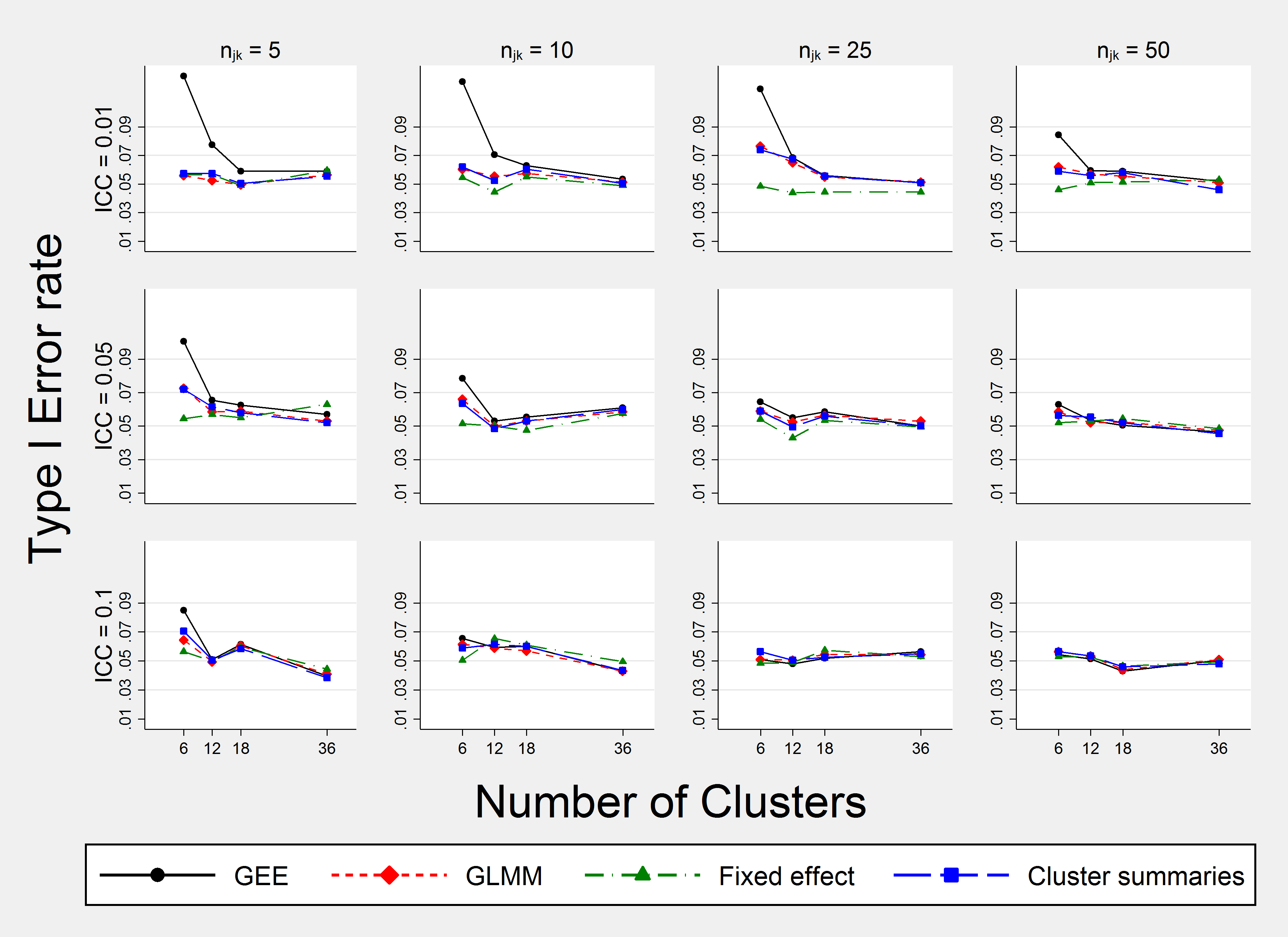

Supplement: Additional file 3: — TIFF image, LZW compression. Type I error rate in the intervention effect estimate $\left({\widehat{\mathit{\ss}}}_1\right)$ß^1 for models that correctly adjust for time. Estimates are obtained from fitting models (1) to (4). Simulated data have six steps, a cell size equal to n jk, a true intervention effect odds ratio of 1 and a time effect odds ratio of 1.03. (TIF 279 kb) [file 13063_2017_1862_MOESM3_ESM.tif]
