# Supplementary material for: Minimum number of clusters and comparison of analysis methods for cross sectional stepped wedge cluster randomised trials with binary outcomes: A simulation study
Source: Trials. 2017 Mar 9;18:119. doi: 10.1186/s13063-017-1862-2 (PMC5345156; doi:10.1186/s13063-017-1862-2)
Supplement: Additional file 4: — Power to detect intervention effect (OR = 1.33) in a six-step SW-CRT with different methods of analysis. Each estimate is based on 2000 simulations. All methods adjust for time in the model. (RTF 153 kb) [file 13063_2017_1862_MOESM4_ESM.rtf]

	True time effect OR = 1.03	
ICC	k	njk	GEE	GLMM	Cluster Summaries
Method	Fixed Effects
Model	
0.01	6	5	0.166	0.094	0.099	0.077	
		10	0.217	0.155	0.153	0.110	
		25	0.309	0.266	0.260	0.202	
		50	0.427	0.404	0.391	0.358	
	12	5	0.171	0.146	0.145	0.104	
		10	0.259	0.240	0.243	0.182	
		25	0.440	0.425	0.430	0.341	
		50	0.679	0.673	0.662	0.596	
	18	5	0.200	0.184	0.187	0.134	
		10	0.340	0.323	0.324	0.245	
		25	0.595	0.582	0.583	0.460	
		50	0.851	0.849	0.845	0.800	
	36	5	0.335	0.325	0.326	0.243	
		10	0.560	0.558	0.558	0.426	
		25	0.871	0.871	0.871	0.795	
		50	0.981	0.983	0.981	0.968	
							
0.05	6	5	0.140	0.107	0.099	0.076	
		10	0.155	0.130	0.130	0.106	
		25	0.235	0.223	0.210	0.204	
		50	0.363	0.356	0.337	0.343	
	12	5	0.147	0.133	0.136	0.128	
		10	0.177	0.171	0.175	0.157	
		25	0.363	0.360	0.354	0.340	
		50	0.599	0.602	0.581	0.591	
	18	5	0.173	0.166	0.163	0.143	
		10	0.246	0.245	0.248	0.217	
		25	0.469	0.462	0.454	0.438	
		50	0.767	0.767	0.749	0.753	
	36	5	0.270	0.268	0.272	0.237	
		10	0.427	0.423	0.423	0.383	
		25	0.793	0.792	0.790	0.777	
		50	0.967	0.969	0.963	0.963	
							
0.1	6	5	0.106	0.093	0.094	0.079	
		10	0.114	0.105	0.109	0.105	
		25	0.169	0.172	0.167	0.176	
		50	0.323	0.323	0.298	0.309	
	12	5	0.122	0.117	0.120	0.115	
		10	0.172	0.164	0.164	0.158	
		25	0.322	0.320	0.312	0.308	
		50	0.557	0.567	0.541	0.559	
	18	5	0.137	0.130	0.136	0.111	
		10	0.244	0.242	0.239	0.227	
		25	0.460	0.461	0.451	0.460	
		50	0.743	0.740	0.723	0.732	
	36	5	0.235	0.233	0.232	0.214	
		10	0.383	0.385	0.378	0.370	
		25	0.745	0.738	0.732	0.732	
		50	0.960	0.959	0.952	0.957	
