# Supplementary material for: Minimum number of clusters and comparison of analysis methods for cross sectional stepped wedge cluster randomised trials with binary outcomes: A simulation study
Source: Trials. 2017 Mar 9;18:119. doi: 10.1186/s13063-017-1862-2 (PMC5345156; doi:10.1186/s13063-017-1862-2)
Supplement: Additional file 6: — Simulation code for scenario B. (DOCX 22 kb) [file 13063_2017_1862_MOESM6_ESM.docx]

**%MACRO** SimbinarySWRCT6(Outset, seed1, NReps, nclusters, nkperstep, njk, pbl, IntEffectOR, timeeffectOR, ICC, output);

ods noresults;

proc printto log = "C:\Users\xxxx\Desktop\Log Files\&output.log20%vs25%.txt"; run;

* Generation of data;

%let rep = 1;

%let truerep = 1;

%do %while (&rep <= &NReps.);

Data Temp;

rep = &rep;

truerep = &truerep;

seed = &seed1. + &truerep.;

call streaminit(seed);

Ysum = **0**;

do k = **1** to &nclusters.; /* Simulate clusters */

pk = RAND('BETA', &pbl. * ((**1**/&ICC.)-**1**), (**1** - &pbl.) * ((**1**/&ICC.)-**1**));

do j = **0** to **6**; /* Simulate timepoints */

if j = **0** then intervention = **0**;

else if j = **1** & k <= &nkperstep. then intervention = **1**;

else if j = **1** & k > &nkperstep. then intervention = **0**;

else if j = **2** & k <= **2** * &nkperstep. then intervention = **1**;

else if j = **2** & k > **2** * &nkperstep. then intervention = **0**;

else if j = **3** & k <= **3** * &nkperstep. then intervention = **1**;

else if j = **3** & k > **3** * &nkperstep. then intervention = **0**;

else if j = **4** & k <= **4** * &nkperstep. then intervention = **1**;

else if j = **4** & k > **4** * &nkperstep. then intervention = **0**;

else if j = **5** & k <= **5** * &nkperstep. then intervention = **1**;

else if j = **5** & k > **5** * &nkperstep. then intervention = **0**;

else if j = **6** then intervention = **1**;

timeeffectOR = &timeeffectOR.;

IntEffectOR = &IntEffectOR.;

logit_pjk = log(pk/(**1**-pk)) + log(IntEffectOR)*intervention + log(timeeffectOR)*j;

pjk = exp(logit_pjk) / (**1** + exp(logit_pjk));

do n = **1** to &njk.; /* Simulate n observations per cluster per time */

Yijk = RAND('BINOMIAL', pjk, **1**);

output; /* output observations to outset */

Ysum = Ysum + Yijk;

if k = &nclusters. & n = &njk. then call symputx('Ysum', Ysum);

end;

end;

end;

RUN;

%let truerep = %eval(&truerep + 1);

%if &Ysum = **0** %then %do;

%let rep = %eval(&rep + 0);

PROC DATASETS noprint;

Delete Temp;

RUN; QUIT;

%end;

%else %do;

%if &rep = **1** %then %do;

Data &Outset.; set Temp; RUN;

%end;

%else %do;

Data &Outset. (drop = truerep seed Ysum pk timeeffectOR IntEffectOR logit_pjk); set &Outset. Temp; RUN;

%end;

%let rep = %eval(&rep + 1);

%end;

%end;

* Checking Design Matrix;

PROC SORT data = &Outset.; BY Rep k j; RUN;

PROC SUMMARY data = &Outset.;

Where Rep = **1**;

Var intervention;

BY k j;

Output Out = DMat;

RUN;

Data DMat; set DMat; IF _STAT_ = "MEAN"; RUN;

PROC SORT data = DMat; BY k;

PROC TRANSPOSE data = DMat Out = ObsMatrix (drop = _NAME_) prefix = j;

Var _FREQ_;

id j;

BY k;

RUN;

PROC TRANSPOSE data = DMat Out = DesignMatrix (drop = _NAME_) prefix = j;

Var intervention;

id j;

BY k;

RUN;

Title "Observation Matrix";

PROC PRINT data = ObsMatrix noobs; RUN;

Title "Design Matrix";

PROC PRINT data = DesignMatrix noobs; RUN;

goptions reset = all;

* GEE Model with time effect, expressed as Odds Ratio;

ods select none;

PROC GENMOD data = &Outset. descending;

ods output GEEEmpPEst = TempOR_RVE_Time (Keep = Rep Parm Estimate ProbZ rename = (Parm = Effect Estimate = OR_RVE_Time ProbZ = P_OR_RVE_Time))

GEEModPEst = TempOR_EXCH_Time (Keep = Rep Parm Estimate ProbZ rename = (Parm = Effect Estimate = OR_EXCH_Time ProbZ = P_OR_EXCH_Time))

GEEExchCorr = ICC_OR_Time (Keep = Rep nValue1 rename = (nValue1 = ICC_OR_Time));

class k;

Model Yijk = j intervention / dist = binomial link = logit;

Repeated subject = k / Type = EXCH ModelSE;

BY Rep;

RUN;

* GEE Model with time effect, expressed as Odds Ratio using the KC correction;

ods select none;

PROC GLIMMIX data = &Outset. Empirical = ROOT;

ods output ParameterEstimates = TempOR_KC_Time (Keep = Rep Effect Estimate Probt rename = (Estimate = OR_KC_Time Probt = P_OR_KC_Time));

class k;

Model Yijk = j intervention / dist = binomial link = logit Solution CL;

Random _residual_ / subject=k type=cs vcorr;

BY Rep;

RUN;

* GEE Model without time effect, expressed as Odds Ratio;

PROC GENMOD data = &Outset. descending;

ods output GEEEmpPEst = TempOR_RVE_Notime (Keep = Rep Parm Estimate ProbZ rename = (Parm = Effect Estimate = OR_RVE_Notime ProbZ = P_OR_RVE_Notime))

GEEModPEst = TempOR_EXCH_Notime (Keep = Rep Parm Estimate ProbZ rename = (Parm = Effect Estimate = OR_EXCH_Notime ProbZ = P_OR_EXCH_Notime))

GEEExchCorr = ICC_OR_Notime (Keep = Rep nValue1 rename = (nValue1 = ICC_OR_Notime));

class k;

Model Yijk = intervention / dist = binomial link = logit;

Repeated subject = k / Type = EXCH ModelSE;

BY Rep;

RUN;

* Fixed Effects model with time effect, expressed as Odds Ratio;

PROC GENMOD data = &Outset. descending;

ods output ParameterEstimates = TempOR_Fixed_Time (Keep = Rep Parameter Estimate ProbChiSq rename = (Parameter = Effect Estimate = OR_Fixed_Time ProbChiSq = P_OR_Fixed_Time));

class k;

Model Yijk = k j intervention / dist = binomial link = logit;

BY Rep;

RUN;

* Fixed Effects model without time effect, expressed as Odds Ratio;

PROC GENMOD data = &Outset. descending;

ods output ParameterEstimates = TempOR_Fixed_Notime (Keep = Rep Parameter Estimate ProbChiSq rename = (Parameter = Effect Estimate = OR_Fixed_Notime ProbChiSq = P_OR_Fixed_Notime));

class k;

Model Yijk = k intervention / dist = binomial link = logit;

BY Rep;

RUN;

* Random Effects model with time effect, expressed as Odds Ratio;

PROC GLIMMIX data = &Outset. method = quad (qpoints = **4**);

ods output ParameterEstimates = TempOR_Mixed_Time (Keep = Rep Effect Estimate Probt rename = (Estimate = OR_Mixed_Time Probt = P_OR_Mixed_Time));

class k;

Model Yijk (descending) = j intervention / dist = binomial link = logit Solution CL;

Random intercept / subject = k;

BY Rep;

RUN;

* Random Effects model without time effect, expressed as Odds Ratio;

PROC GLIMMIX data = &Outset. method = quad (qpoints = **4**);

ods output ParameterEstimates = TempOR_Mixed_Notime (Keep = Rep Effect Estimate Probt rename = (Estimate = OR_Mixed_Notime Probt = P_OR_Mixed_Notime));

class k;

Model Yijk (descending) = intervention / dist = binomial link = logit Solution CL;

Random intercept / subject = k;

BY Rep;

RUN;

* Cluster Summaries Method (Hussey and Hughes) with time effect, expressed as Risk Difference;

PROC SUMMARY data = &Outset.;

Var Yijk;

output out = tempsummary (drop = _TYPE_ _FREQ_) mean = p;

BY Rep k j;

RUN;

PROC SORT data = tempsummary; BY k j; RUN;

Data tempsum2 (Drop = _TYPE_ _FREQ_ _STAT_); merge tempsummary Dmat; BY k j; RUN;

PROC SORT data = tempsum2; BY Rep k j; RUN;

PROC MIXED data = tempsum2;

ods output SolutionF = TempRD_CSum_Time (Keep = Rep Effect Estimate Probt rename = (Estimate = RD_CSum_Time probt = P_RD_CSum_Time));

class k;

Model p = j intervention / Solution CL;

Random Intercept / Subject = k;

BY Rep;

RUN;

* Cluster Summaries Method (Hussey and Hughes) without time effect, expressed as Risk Difference;

PROC MIXED data = tempsum2;

ods output SolutionF = TempRD_CSum_Notime (Keep = Rep Effect Estimate Probt rename = (Estimate = RD_CSum_Notime probt = P_RD_CSum_Notime));

class k;

Model p = intervention / Solution CL;

Random Intercept / Subject = k;

BY Rep;

RUN;

* Checking Model Power and Bias;

PROC SORT data = TempOR_RVE_Time; BY Rep Effect; RUN;

PROC SORT data = TempOR_EXCH_Time; BY Rep Effect; RUN;

PROC SORT data = TempOR_KC_Time; BY Rep Effect; RUN;

PROC SORT data = TempOR_RVE_Notime; BY Rep Effect; RUN;

PROC SORT data = TempOR_EXCH_Notime; BY Rep Effect; RUN;

PROC SORT data = TempOR_Fixed_Time; BY Rep Effect; RUN;

PROC SORT data = TempOR_Fixed_Notime; BY Rep Effect; RUN;

PROC SORT data = TempOR_Mixed_Time; BY Rep Effect; RUN;

PROC SORT data = TempOR_Mixed_Notime; BY Rep Effect; RUN;

PROC SORT data = TempRD_CSum_Time; BY Rep Effect; RUN;

PROC SORT data = TempRD_CSum_Notime; BY Rep Effect; RUN;

Data temp&output.; Merge TempOR_RVE_Time TempOR_EXCH_Time TempOR_KC_Time

TempOR_RVE_Notime TempOR_EXCH_Notime

TempOR_Fixed_Time TempOR_Fixed_Notime

TempOR_Mixed_Time TempOR_Mixed_Notime

TempRD_CSum_Time TempRD_CSum_Notime;

BY Rep Effect;

IF Effect = "intervention";

ARRAY A(*) P_OR_RVE_Time P_OR_EXCH_Time P_OR_KC_Time P_OR_RVE_Notime P_OR_EXCH_Notime

P_OR_Fixed_Time P_OR_Fixed_Notime

P_OR_Mixed_Time P_OR_Mixed_Notime

P_RD_CSum_Time P_RD_CSum_Notime;

ARRAY B(*) ORRVETime_Power OREXCHTime_Power ORKCTime_Power ORRVENotime_Power OREXCHNotime_Power

ORFixedTime_Power ORFixedNotime_Power

ORMixedTime_Power ORMixedNotime_Power

RDCSumTime_Power RDCSumNotime_Power;

ARRAY C(*) ORRVETime_Failed OREXCHTime_Failed ORKCTime_Failed ORRVENotime_Failed OREXCHNotime_Failed

ORFixedTime_Failed ORFixedNotime_Failed

ORMixedTime_Failed ORMixedNotime_Failed

RDCSumTime_Failed RDCSumNotime_Failed;

do i = **1** to Dim(A);

IF **.** < A[i] < **0.05** then B[i] = **1**;

ELSE IF A[i] >= **0.05** then B[i] = **0**;

ELSE IF A[i] = **.** then B[i] = **.**;

IF A[i] = **.** then C[i] = **1**;

ELSE IF A[i] ~= **.** then C[i] = **0**;

end;

TrueEffect_RD = (((&IntEffectOR.*&pbl.)/(**1**-&pbl.)) / (**1** + (&IntEffectOR.*&pbl.)/(**1**-&pbl.))) - &pbl.;

TrueEffect_logOR = log(&IntEffectOR.);

ARRAY OR1(*) OR_RVE_Time OR_EXCH_Time OR_KC_Time OR_RVE_Notime OR_EXCH_Notime

OR_Fixed_Time OR_Fixed_Notime

OR_Mixed_Time OR_Mixed_Notime;

ARRAY OR2(*) ORRVETime_Bias OREXCHTime_Bias ORKCTime_Bias ORRVENotime_Bias OREXCHNotime_Bias

ORFixedTime_Bias ORFixedNotime_Bias

ORMixedTime_Bias ORMixedNotime_Bias;

ARRAY OR3(*) ORRVETime_PctB OREXCHTime_PctB ORKCTime_PctB ORRVENotime_PctB OREXCHNotime_PctB

ORFixedTime_PctB ORFixedNotime_PctB

ORMixedTime_PctB ORMixedNotime_PctB;

do j = **1** to Dim(OR1);

OR2[j] = OR1[j] - TrueEffect_logOR;

OR3[j] = ((OR1[j] - TrueEffect_logOR) / TrueEffect_logOR) * **100**;

end;

ARRAY RD1(*) RD_CSum_Time RD_CSum_Notime;

ARRAY RD2(*) RDCSumTime_Bias RDCSumNotime_Bias;

ARRAY RD3(*) RDCSumTime_PctB RDCSumNotime_PctB;

do k = **1** to Dim(RD1);

RD2[k] = RD1[k] - TrueEffect_RD;

RD3[k] = ((RD1[k] - TrueEffect_RD) / TrueEffect_RD) * **100**;

end;

drop i j k;

length set $50.;

set = "&Output.";

RUN;

PROC SORT data = ICC_OR_Time; BY Rep; RUN;

PROC SORT data = ICC_OR_Notime; BY Rep; RUN;

PROC SORT data = temp&output.; BY Rep; RUN;

Data &output.; Merge temp&output. ICC_OR_Time ICC_OR_Notime; BY Rep; RUN;

* Summary of Results;

PROC SUMMARY data = &output. print;

output out = TempStatSummary (drop = _TYPE_ _FREQ_);

Var ORRVETime_Power OREXCHTime_Power ORKCTime_Power ORRVENotime_Power OREXCHNotime_Power

ORFixedTime_Power ORFixedNotime_Power

ORMixedTime_Power ORMixedNotime_Power

RDCSumTime_Power RDCSumNotime_Power

ORRVETime_Failed OREXCHTime_Failed ORKCTime_Failed ORRVENotime_Failed OREXCHNotime_Failed

ORFixedTime_Failed ORFixedNotime_Failed

ORMixedTime_Failed ORMixedNotime_Failed RDCSumTime_Failed RDCSumNotime_Failed

RDCSumTime_Bias RDCSumNotime_Bias

ORRVETime_Bias OREXCHTime_Bias ORKCTime_Bias ORRVENotime_Bias OREXCHNotime_Bias

ORFixedTime_Bias ORFixedNotime_Bias

ORMixedTime_Bias ORMixedNotime_Bias

RDCSumTime_PctB RDCSumNotime_PctB

ORRVETime_PctB OREXCHTime_PctB ORKCTime_PctB ORRVENotime_PctB OREXCHNotime_PctB

ORFixedTime_PctB ORFixedNotime_PctB

ORMixedTime_PctB ORMixedNotime_PctB;

RUN;

Data TempStatSummary2; set TempStatSummary;

IF _STAT_ = "MEAN";

Drop _STAT_;

RUN;

PROC TRANSPOSE Data = TempStatSummary2 Out = TempStatSummary3; RUN;

Data TempStatPower (Rename = (COL1 = Power)) TempStatBias (Rename = (COL1 = Bias)) TempStatPctB (Rename = (COL1 = PctB))

TempStatFailed (Rename = (COL1 = Failed)); set TempStatSummary3;

IF index(_NAME_, 'Power') > **0** then output TempStatPower;

ELSE IF index(_NAME_, 'Bias') > **0** then output TempStatBias;

ELSE IF index(_NAME_, 'PctB') > **0** then output TempStatPctB;

ELSE IF index(_NAME_, 'Failed') > **0** then output TempStatFailed;

RUN;

Data TempStatPower2 (drop = _NAME_); set TempStatPower;

Statistic = Scan(_NAME_,**1**,'_');

RUN;

Data TempStatBias2 (drop = _NAME_); set TempStatBias;

Statistic = Scan(_NAME_,**1**,'_');

RUN;

Data TempStatPctB2 (drop = _NAME_); set TempStatPctB;

Statistic = Scan(_NAME_,**1**,'_');

RUN;

Data TempStatFailed2 (drop = _NAME_); set TempStatFailed;

Statistic = Scan(_NAME_,**1**,'_');

RUN;

PROC SORT data = TempStatPower2; BY Statistic; RUN;

PROC SORT data = TempStatBias2; BY Statistic; RUN;

PROC SORT data = TempStatPctB2; BY Statistic; RUN;

PROC SORT data = TempStatFailed2; BY Statistic; RUN;

Data Summary&output.; Merge TempStatPower2 TempStatBias2 TempStatPctB2 TempStatFailed2; BY Statistic;

K = &nclusters.;

n = &njk.;

pControl = &pbl.;

EffectOR = &IntEffectOR.;

TimeEffectOR = &timeeffectOR.;

ICC = &ICC.;

RUN;

* Tidy Up;

PROC DATASETS noprint;

Delete &Outset. Temp /*DMat ObsMatrix DesignMatrix*/ ICC_OR_Time ICC_OR_Notime

TempOR_RVE_Time TempOR_EXCH_Time TempOR_KC_Time TempOR_RVE_Notime TempOR_EXCH_Notime

TempOR_Fixed_Time TempOR_Fixed_Notime TempOR_Mixed_Time TempOR_Mixed_Notime

tempsummary tempsum2 TempRD_CSum_Time TempRD_CSum_Notime temp&output.

TempStatSummary TempStatSummary2 TempStatSummary3 TempStatPower TempStatPower2 TempStatBias TempStatBias2 TempStatPctB TempStatPctB2 TempStatFailed TempStatFailed2;

RUN; QUIT;

proc printto; run;

ods results;

**%MEND**;

* SimbinarySWRCT(Outset, seed1, NReps, nclusters, nkperstep, njk, pbl, IntEffect, timeeffect, ICC, output);

*%SimbinarySWRCT6(Outset, 123456, 2000, 18, 3, 25, 0.2, 1.33, 1.03, 0.1, testoutput)
